# Autonomic imbalance and vascular injury in hypertensive chronic kidney disease: mechanisms and clinical potential of ultrasound-guided sympathetic blockade

**DOI:** 10.3389/fnins.2026.1836903

**Published:** 2026-05-26

**Authors:** Xiang Gao, Zhimin Fu, Zeyu Wang, Zhigan Lv, Weiwei Zhang

**Affiliations:** Third Hospital of Shanxi Medical University, Shanxi Bethune Hospital, Shanxi Academy of Medical Sciences, Tongji Shanxi Hospital, Taiyuan, China

**Keywords:** autonomic imbalance, hypertensive chronic kidney disease, neuromodulation, precision medicine, ultrasound-guided sympathetic blockade

## Abstract

Hypertensive Chronic Kidney Disease (CKD) constitutes a significant global health burden, characterized by a vicious cycle of hypertension and progressive renal decline. Autonomic imbalance, specifically sympathetic overactivity and parasympathetic withdrawal, is increasingly recognized as a central driver of this pathophysiology, interacting with traditional hemodynamic factors such as RAAS activation and volume overload. This review aims to elucidate the deep-seated mechanisms by which autonomic imbalance induces vascular injury and renal progression, and to evaluate the clinical potential of ultrasound-guided sympathetic blockade as a novel therapeutic strategy. We conducted a comprehensive narrative review of recent basic research and clinical evidence regarding the “Kidney-Brain-Vascular Axis” and neuromodulation therapies in the context of hypertensive CKD. The pathogenesis involves a maladaptive “Neuro-Immune-Vascular Axis.” Sympathetic overactivity not only induces hemodynamic stress but also disrupts the Th1/Th2 immune balance, accelerating vascular calcification and fibrosis. Ultrasound-guided sympathetic blockade offers a reversible, minimally invasive neuromodulation approach. Preliminary clinical evidence suggests it may lower blood pressure, improves vascular endothelial function, and potentially delays renal progression, particularly in patients with resistant hypertension, with a superior safety profile compared to renal denervation. Ultrasound-guided sympathetic blockade represents an emerging and investigational adjunct, constrained by the lack of large-scale randomized controlled trials and long-term outcome data.

## 1 Introduction

### 1.1 Clinical background and challenges

CKD has emerged as a substantial global public health burden, affecting approximately 10% to 15% of the adult population worldwide (Jha et al., 2013). Hypertension is not only a common manifestation of and comorbidity associated with CKD but also a primary driver of its progression. The relationship between hypertension and CKD is characterized by a bidirectional “vicious cycle”: hypertension accelerates glomerular sclerosis and renal damage, while impaired renal function leads to volume expansion and neurohormonal activation, further exacerbating blood pressure elevation (Shin and Lee, 2021).

Current standard pharmacological therapies, particularly Renin-Angiotensin-Aldosterone System (RAAS) inhibitors such as ACEIs and ARBs, serve as the cornerstone for delaying CKD progression. However, these therapies exhibit significant limitations. While RAS inhibitors effectively reduce proteinuria and slow the decline in glomerular filtration rate (GFR), they fail to completely eliminate the residual risk of cardiovascular events and renal failure (Bakris et al., 2020). Furthermore, a substantial proportion of CKD patients suffer from resistant hypertension (RHTN), defined as blood pressure remaining above target despite the concurrent use of three or more antihypertensive agents from different classes at maximal tolerated doses. The prevalence of RHTN in the CKD population is markedly higher than in the general hypertensive population, ranging from 30% to 50% (Fay and Cohen, 2021). This condition is associated with a significantly worse prognosis, leading to accelerated cardiovascular disease and end-stage renal disease (ESRD), thereby underscoring the urgent need for novel therapeutic strategies.

### 1.2 The central role of the autonomic nervous system in hypertensive CKD

Historically, hypertensive CKD has been conceptualized primarily as a hemodynamic disorder driven by fluid overload and vasoconstriction. However, emerging evidence suggests a paradigm shift, recognizing the condition as a multifactorial disease where autonomic dysfunction is a critical and integrated component, working alongside established drivers such as RAAS activation and endothelial dysfunction. CKD is intrinsically linked to a state of sympathetic overactivity (SOA) and parasympathetic withdrawal, collectively termed autonomic imbalance (Grassi et al., 2015).

The kidney acts not merely as a target of hypertension but as a sensory organ. Renal afferent nerves transmit signals from the diseased kidney to the central nervous system, triggering a reflex increase in central sympathetic outflow (Joles and Koomans, 2004). This sympathetic hyperactivity, in turn, promotes systemic vasoconstriction, sodium retention, and vascular remodeling. Consequently, intervening in this autonomic imbalance represents a promising strategy to disrupt the pathophysiological chain of “hypertension-renal injury-vascular sclerosis,” moving beyond the limitations of conventional hemodynamic control.

### 1.3 Purpose of this review

Given the pivotal role of autonomic dysfunction in the pathogenesis of hypertensive CKD, this review aims to: (1) elucidate the deep-seated mechanisms by which autonomic imbalance leads to vascular injury and renal progression; and (2) evaluate the clinical potential of ultrasound-guided sympathetic blockade as a novel, minimally invasive, and reversible intervention for managing RHTN in CKD patients, thereby offering a therapeutic target distinct from traditional pharmacology.

### 1.4 Methodology and search strategy

To ensure a comprehensive and transparent review, a structured literature search was conducted using PubMed, Scopus, and Web of Science databases up to December 2023. Search terms included combinations of “Hypertension,” “Chronic Kidney Disease,” “Autonomic Nervous System,” “Sympathetic Overactivity,” “Ultrasound-guided,” “Sympathetic Blockade,” and “Neuromodulation.” Studies were included if they provided mechanistic insights into the autonomic-renal axis or presented clinical data on ultrasound-guided sympathetic blockade in hypertensive or CKD populations. Non-English articles, case reports lacking mechanistic data, and studies focusing exclusively on pharmacological therapy without a neuromodulatory component were excluded.

## 2 Autonomic dysfunction in hypertensive CKD: from mechanisms to effects

The intersection of CKD and hypertension represents a clinical conundrum underpinned by a multifactorial pathophysiology, where a maladaptive autonomic nervous system (ANS) acts as a critical amplifier alongside RAAS activation and volume overload. The dysregulation, characterized by sympathetic overactivity and parasympathetic withdrawal, is not merely a consequence of renal impairment but a potent driver of disease progression. To comprehensively elucidate this process, we introduce the “Kidney–Brain–Vascular Axis” as a core theoretical framework (Grassi et al., 2015; Joles and Koomans, 2004). This axis conceptualizes the dynamic interplay where renal injury triggers central sympathetic activation, which in turn precipitates systemic vascular damage, creating a self-perpetuating pathogenic loop. This section elucidates the pathogenic origins of this autonomic imbalance and details the downstream vascular consequences that exacerbate the cardiorenal syndrome, incorporating evidence from basic research to validate these mechanisms. We specifically distinguish between associative observations and established causal mechanisms, and differentiate findings derived from experimental models versus those supported by clinical evidence to avoid an overly confirmatory tone.

### 2.1 Pathological origins of autonomic imbalance

The genesis of autonomic dysfunction in hypertensive CKD is multifactorial, involving a complex interplay between central neural reprogramming, renal afferent signaling, and the systemic uremic milieu.

1. *Central mechanisms*: At the core of central sympathetic dysregulation lies the rostral ventrolateral medulla (RVLM), the primary vasomotor center. In hypertensive CKD models, RVLM neurons exhibit aberrant excitability, often driven by angiotensin II (Ang II) signaling and oxidative stress (Elsaafien et al., 2021). Basic research utilizing animal models has supported a causal role for this central mechanism in experimental settings; for instance, Gao et al. (2007) demonstrated in heart failure rabbits that exercise training normalizes AT1R signaling in the RVLM and reduces ROS production, thereby attenuating sympathetic outflow. Conversely, microinjection of Ang II or ROS donors into the RVLM in rodent models directly potentiates the firing rate of pre-sympathetic neurons, mimicking the hypertensive state. Concurrently, the baroreflex mechanism, which buffers blood pressure fluctuations, is compromised. In CKD, the arterial baroreflex is reset to maintain a higher blood pressure threshold, and its sensitivity is significantly attenuated. This baroreflex resetting reduces the inhibitory brake on sympathetic outflow, creating a permissive environment for sustained hypertension (Grassi et al., 2011). Furthermore, central inflammation within the hypothalamus and RVLM, marked by microglial activation and pro-inflammatory cytokine release, directly stimulates sympathetic output and contributes to neurogenic hypertension (Shi et al., 2010). Additionally, impairment of the GABAergic inhibitory system—specifically reduced GABAergic tone and plasticity of GABAergic synapses in the hypothalamus and RVLM—disinhibits pre-sympathetic neurons, further exacerbating central sympathetic drive (Li et al., 2008).
2. *Renal local neural feedback*: The kidney is not merely a target of sympathetic innervation but an active contributor to autonomic dysregulation via the renorenal reflex. Ischemic nephrons, a hallmark of hypertensive nephropathy, activate renal chemoreceptors and mechanoreceptors. These signals are transmitted via renal afferent nerves to the central nervous system, specifically integrating into the hypothalamus and RVLM. Specifically, endothelin B (ETB) receptor deficiency in the renal vasculature has been identified as a critical mechanism elevating afferent renal nerve activity, thereby contributing to neurogenic hypertension (Becker et al., 2023). Landmark studies by Campese et al. (2006) provided crucial mechanistic proof in animal models. They demonstrated that renal injury in rats activates this afferent pathway, triggering a sustained rise in blood pressure. Crucially, this hypertensive response was abrogated by renal denervation or dorsal rhizotomy, confirming that the ‘kidney-to-brain’ signal transmission is essential for the maintenance of neurogenic hypertension. This afferent signaling triggers a reflex increase in efferent sympathetic nerve activity (SNA) to the kidneys and other vascular beds, establishing a vicious cycle of neurogenic hypertension.
3. *Uremic toxins and inflammation*: The systemic environment in CKD further amplifies sympathetic drive. The accumulation of uremic toxins stimulates peripheral chemoreceptors, particularly the carotid body. Animal experiments have validated this pathway; studies in chronic renal failure (CRF) rats have shown that the carotid body exhibits exaggerated chemoreflex sensitivity. Furthermore, the micro-inflammatory state characteristic of CKD—marked by elevated cytokines such as TNF-α and IL-6—contributes to autonomic imbalance (Stenvinkel, 2010) highlighted that oxidative stress and inflammation in the central nervous system, driven by uremic toxins, directly stimulate central sympathetic output. This toxic-inflammatory milieu creates a positive feedback loop where inflammation begets sympathetic activation, which in turn promotes further inflammation. Furthermore, emerging evidence highlights the role of the “Gut-Brain-Kidney Axis,” where autonomic dysfunction exacerbates gut dysbiosis and systemic inflammation, mediated in part by short-chain fatty acid receptors (Xu et al., 2022).

### 2.2 Deep mechanisms of vascular injury induced by autonomic imbalance

**Figure 1:**
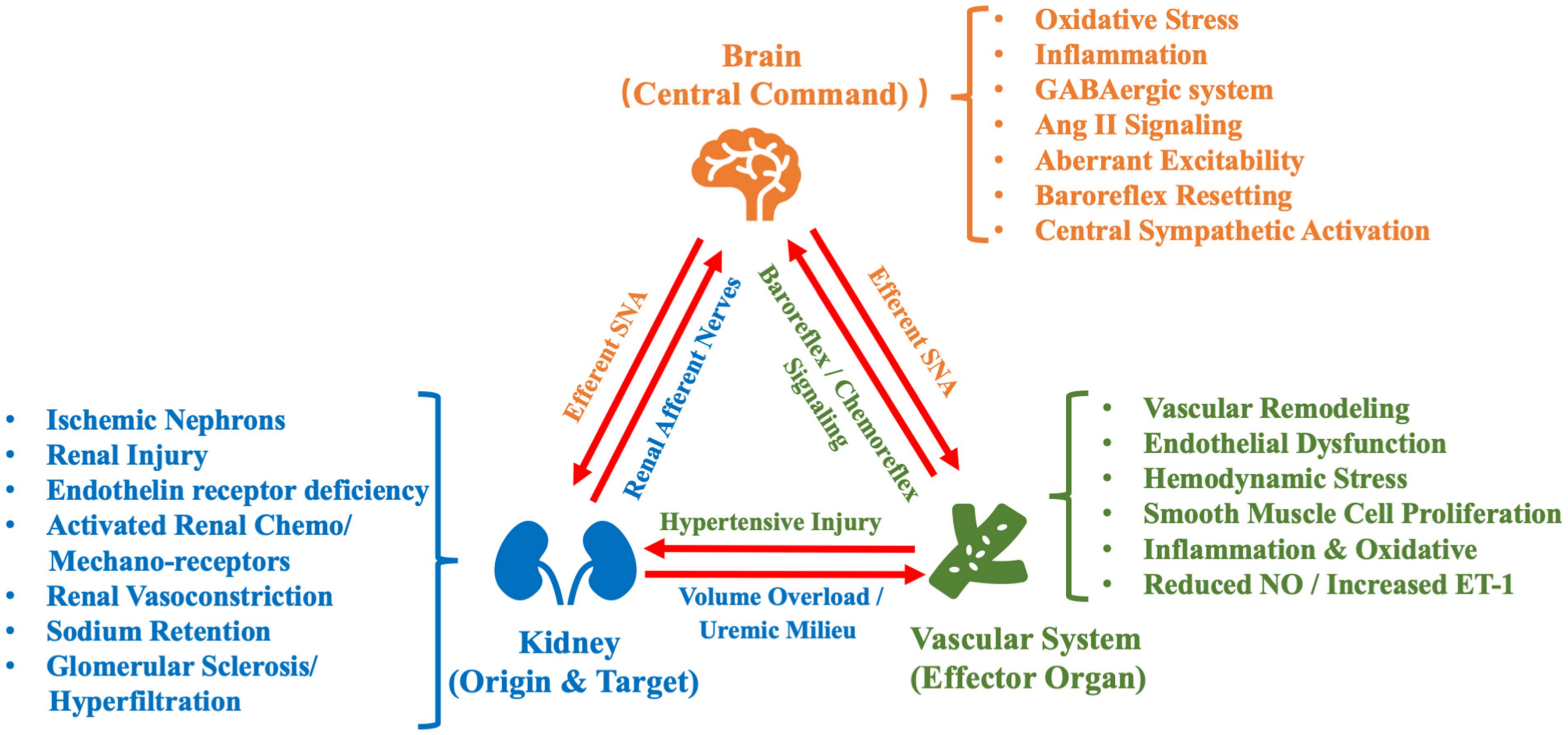
Pathological mechanisms of the “kidney-brain-vascular axis.” At the top lies the brain (central command), where aberrant excitability, oxidative stress, central inflammation, and impaired GABAergic inhibition in the RVLM and hypothalamus trigger central sympathetic activation and baroreflex resetting. The kidney serves as both the origin and target: injured nephrons—including those affected by ET_B_ receptor deficiency—send afferent signals to the brain that sustain sympathetic drive, while receiving efferent signals that cause vasoconstriction and sodium retention. The vascular/arteriolar system acts as the effector organ, responding to sympathetic output with endothelial dysfunction, remodeling, and atherosclerosis. These three components form a self-perpetuating vicious cycle: the brain drives vascular and renal damage, vascular stress inflicts renal injury, and renal pathology further disrupts vascular function, collectively accelerating the progression of hypertension.

The chronic sympathetic overdrive observed in hypertensive CKD precipitates profound structural and functional vascular alterations, accelerating morbidity and mortality (Figure 1).

1. *Hemodynamic effects*: The immediate hemodynamic consequence of sympathetic hyperactivation is the increased release of norepinephrine (NE) from postganglionic nerve endings. NE binds to adrenergic receptors on vascular smooth muscle cells (VSMCs), inducing vasoconstriction. This elevates peripheral vascular resistance and systemic blood pressure. Crucially, the sustained increase in pressure and flow generates abnormal shear stress on the endothelial surface, which acts as a mechanical trigger for vascular remodeling (Schiffrin, 2012).
2. *Smooth muscle cell proliferation*: Beyond acute contraction, chronic NE exposure induces structural remodeling. Evidence from animal models suggests this causality in specific experimental contexts: chronic infusion of NE in rats induces VSMC hypertrophy and hyperplasia (hypercellularity) similar to that seen in hypertension, while sympathetic denervation in hypertensive animal models has been shown to regress vascular medial thickness (Folkow, 1982; Elsaafien et al., 2021). The activation of alpha-1-adrenergic receptors on VSMCs stimulates hypertrophic signaling pathways, including the mitogen-activated protein kinase (MAPK) cascade. This leads to VSMC hypertrophy and hyperplasia, contributing to the thickening of the arterial media and reduced lumen diameter (Intengan and Schiffrin, 2001). Recent insights from Elsaafien have further elucidated the neurovascular cross-talk in this process, highlighting that autonomic dysregulation directly modulates the phenotypic switching of VSMCs via specific neurogenic signals (Elsaafien et al., 2026). While these animal studies establish a plausible biological mechanism, translational clinical data demonstrating direct causality in human hypertensive CKD remains less definitive, often relying on associative evidence.
3. *Endothelial dysfunction*: The endothelium is a primary casualty of autonomic imbalance. Excessive sympathetic activity reduces the bioavailability of nitric oxide (NO), the principal vasodilator, partly through NE-induced ROS production which scavenges NO. Simultaneously, sympathetic activation stimulates the release of endothelin-1 (ET-1), a potent vasoconstrictor and mitogen. Animal studies in Sprague–Dawley rats have demonstrated that renal denervation can restore endothelial function by increasing endothelial nitric oxide synthase (eNOS) expression and reducing oxidative stress markers in the aorta, supporting a direct link between sympathetic tone and endothelial integrity in preclinical models (Higashi et al., 2001; Schiffrin, 2001; Damascelli et al., 2013). The imbalance between reduced NO and increased ET-1 impairs vasodilatory capacity and promotes a pro-thrombotic state (Dinh et al., 2014).
4. *Matrix deposition*: Sympathetic tone influences the vascular extracellular matrix. Activated fibroblasts and increased collagen deposition within the adventitia and media reduce vascular compliance. This stiffening is a critical factor in isolated systolic hypertension, prevalent in CKD patients.
5. *Inflammation and oxidative stress pathways*: Sympathetic neurotransmitters act as immunomodulators. NE can activate the NF-κB pathway in vascular cells and infiltrating immune cells. *In vivo* animal experiments have shown that chemical sympathectomy in hypertensive models reduces the infiltration of macrophages and T-cells into the vascular wall and downregulates adhesion molecules (e.g., VCAM-1, ICAM-1), thereby attenuating vascular inflammation. This vascular inflammation accelerates the progression of atherosclerosis, a process highly prevalent and aggressive in the CKD population (Harrison et al., 2010; Montuoro et al., 2025).
6. *Renal-specific injury*: Finally, efferent sympathetic nerves directly innervate the renal vasculature and tubules. Heightened efferent SNA causes constriction of afferent and efferent arterioles, albeit with a proportionally greater effect on the efferent side. This leads to increased intraglomerular pressure and hyperfiltration. While initially compensatory, chronic hyperfiltration precipitates glomerular hypertrophy and segmental sclerosis. Supportive evidence comes from diabetic and hypertensive rat models, where renal denervation has been shown to reduce proteinuria and glomerular sclerosis, directly linking sympathetic overactivity to renal structural damage (Katholi et al., 1981; Johns, 2014). This accelerates the decline of renal function and perpetuating the cycle of hypertension and kidney damage. These maladaptive vascular changes underscore the need for interventions that directly target the neural drivers of injury, such as ultrasound-guided sympathetic blockade (Hostetter et al., 1981; Vink et al., 2013).

## 3 Ultrasound-guided sympathetic nerve block: technical principles and Neuromodulatory mechanisms

Sympathetic nerve blockade has long been a cornerstone in managing neuropathic pain and vascular disorders. Traditionally performed using surface anatomical landmarks or fluoroscopic guidance, the advent of ultrasound technology has revolutionized these procedures. This section elucidates the technical advantages of ultrasound guidance, the anatomical basis of common blockade targets, and the underlying physiological mechanisms through which these interventions confer vascular protection.

### 3.1 Precision advantages of ultrasound technology

The integration of ultrasound into clinical practice has significantly enhanced the safety and efficacy of sympathetic nerve blocks. The primary advantage lies in the real-time visualization of neural structures and the surrounding vascular anatomy. Unlike fluoroscopy, which primarily visualizes bony landmarks, ultrasound allows for the direct identification of soft tissues, including the sympathetic ganglia, adjacent vessels, and fascial planes (Li et al., 2025). This capability is crucial for avoiding vital structures such as the vertebral artery during stellate ganglion block (SGB) or the aorta during celiac plexus block.

Furthermore, ultrasound guidance has been demonstrated to improve the success rate of puncture while substantially reducing the incidence of complications. By visualizing the needle trajectory in real-time, clinicians can avoid inadvertent vascular puncture, thereby minimizing the risk of hematoma formation. Similarly, the proximity of the sympathetic chain to the pleura (in the thoracic region) or the neuraxis necessitates precise needle placement to prevent catastrophic complications such as pneumothorax or spinal cord injury (Narouze, 2010). Real-time visualization is indispensable for minimizing procedure-related adverse events, noting a significant reduction in inadvertent nerve injury and intravascular injection compared to landmark-based methods. Specific to SGB, ultrasound guidance helps prevent recurrent laryngeal nerve block and brachial plexus block, which are common adverse effects associated with the blind landmark technique (Li et al., 2023).

Another critical benefit is the ability to visualize the diffusion of the local anesthetic. Color Doppler imaging can confirm the distribution of the injectate around the target ganglion, ensuring that the therapeutic agent adequately bathes the neural tissue. This confirmation not only predicts the efficacy of the block but also prevents the wasted deposition of medication into adjacent muscular or vascular compartments, ensuring a more consistent clinical outcome.

### 3.2 Common blockade targets and anatomical basis

1. *Stellate Ganglion Block (SGB)*: The stellate ganglion, formed by the fusion of the inferior cervical ganglion and the first thoracic ganglion, serves as the primary sympathetic relay for the head, neck, and upper extremities. Ultrasound-guided SGB is typically performed at the level of the C6 or C7 vertebra, targeting the pre-vertebral fascia. By interrupting the sympathetic outflow to the upper body, SGB induces vasodilation in the carotid and vertebral arteries, as well as the peripheral vessels of the upper limbs. Beyond regional effects, SGB is recognized for its role in modulating systemic autonomic balance, potentially stabilizing the ANS by reducing sympathetic tone, which has implications for conditions ranging from complex regional pain syndrome (CRPS) to post-traumatic stress disorder (PTSD) (Mulvaney et al., 2010).
2. *Lumbar Sympathetic Block (LSB)*: The lumbar sympathetic chain, typically located anterolateral to the lumbar vertebral bodies, provides sympathetic innervation to the lower extremities and the renal vascular bed. LSB is indicated for patients with lower limb ischemia, such as those suffering from Buerger’s disease or diabetic foot ulcers. From a neuroanatomical perspective, blocking the L2 and L3 ganglia effectively interrupts vasoconstrictive signals to the lower limbs. Moreover, LSB can directly influence renal hemodynamics; by attenuating renal sympathetic nerve activity, the procedure can improve renal blood flow and potentially assist in managing RHTN or renal ischemia (Ayad et al., 2024).
3. *Neurolytic Celiac Plexus Block (NCPB)*: The celiac plexus, situated around the origin of the celiac trunk and superior mesenteric artery, regulates sympathetic supply to the abdominal viscera, including the liver, pancreas, and stomach. NCPB is frequently utilized for palliative pain control in upper abdominal malignancies (Sachdev and Gress, 2018). However, its role in vascular regulation is equally significant. By blocking sympathetic input to the splanchnic vascular bed, NCPB can induce significant vasodilation in the mesenteric and hepatic arteries, improving perfusion to abdominal organs and reducing the vascular resistance associated with chronic visceral ischemia (Richter, 2016).

### 3.3 Physiological mechanisms of neuromodulation in vascular protection

1. *Downregulation of sympathetic tone*: The fundamental mechanism of sympathetic blockade involves the interruption of preganglionic and postganglionic sympathetic fibers. This inhibition effectively decouples the central sympathetic drive from peripheral effectors. Consequently, there is a marked reduction in the release of NE at the neuroeffector junctions within vascular smooth muscle. The decrease in circulating and local NE levels alleviates vasospasm and reduces peripheral vascular resistance, thereby establishing a favorable environment for tissue perfusion (Cousins et al., 2012; McMahon et al., 2013).
2. *Improvement of microcirculation*: By blocking sympathetic vasoconstrictive tone, these interventions relieve arterial spasm and open precapillary sphincters. This leads to a significant increase in capillary perfusion pressure and blood flow velocity. Enhanced microcirculation facilitates the delivery of oxygen and essential nutrients to the vascular endothelium, which is critical for endothelial cell survival and function. This improved endothelial nutrition helps restore vascular barrier integrity and promotes angiogenesis in ischemic tissues (Chien, 2007; Carmeliet, 2000).
3. *Interruption of nociceptive transmission*: Pain and sympathetic activity share a complex, reciprocal relationship (Janig and Baron, 2003; Roberts, 1986). Nociceptive stimuli activate the sympathetic-adrenal axis, leading to the release of catecholamines and neuropeptides, which in turn sensitize peripheral nociceptors and induce vasoconstriction, creating a vicious cycle known as the “pain-vasospasm loop” (Treede et al., 1992). Sympathetic nerve blocks disrupt this pathological feedback loop (Raja and Grabow, 2002). By blocking nociceptive signal transmission and reducing sympathetic efferent activity, the procedure breaks the cycle of pain and vascular spasm, thereby alleviating ischemia and reducing tissue metabolic demand (Janig and Baron, 2003; Stanton-Hicks et al., 2002).
4. *Immunomodulation via the cholinergic anti-inflammatory pathway (CAP)*: Emerging evidence suggests that sympathetic neuromodulation exerts potent anti-inflammatory effects, primarily through the activation of the CAP. While SGB inhibits the sympathetic “fight or flight” response, it may indirectly unmask or enhance parasympathetic activity. This modulation leads to the release of acetylcholine (ACh) from the vagus nerve and other cholinergic fibers. ACh interacts with the *α*7 nicotinic acetylcholine receptor (α7nAChR) on macrophages and other immune cells, inhibiting the release of pro-inflammatory cytokines such as TNF-α, IL-1β, and IL-6. This systemic and local anti-inflammatory effect attenuates vascular wall inflammation, a key driver of atherosclerosis and endothelial dysfunction (Tracey, 2007). Thus, sympathetic blockade offers a dual benefit: restoring vascular hemodynamics and suppressing the inflammatory milieu that threatens vascular integrity.

## 4 Clinical evidence: application of ultrasound-guided sympathetic nerve block in hypertensive CKD

The intersection of interventional pain management and nephrology has garnered increasing attention, particularly regarding the modulation of the sympathetic nervous system (SNS) in CKD patients with hypertension. Ultrasound-guided sympathetic nerve blocks, predominantly SGB and LSB, have evolved from purely analgesic procedures to potential modulators of cardiovascular and renal hemodynamics. However, from an evidence-based medicine perspective, the overall certainty of evidence regarding SGB/LSB for hypertensive CKD warrants cautious interpretation. According to the Grading of Recommendations Assessment, Development and Evaluation (GRADE) system, the current evidence level is generally rated as low to moderate, primarily due to the predominance of observational studies and methodological constraints in available randomized trials (Guyatt et al., 2008). Consequently, ultrasound-guided sympathetic blockade should be considered an emerging and investigational strategy rather than a standard clinical solution at this stage.

### 4.1 Analysis of evidence-based medical evidence

Current evidence suggests that SGB can modulate autonomic balance and hemodynamics, including blood pressure variability (BPV), though data specifically in hypertensive CKD populations remain extremely limited. In non-CKD settings, small-scale RCTs have demonstrated that SGB attenuates cardiovascular stress responses and significantly alters heart rate variability during surgical procedures (Chen et al., 2015; Jiang et al., 2025). Furthermore, in a placebo-controlled, double-blind trial based on a within-subject design, demonstrated that SGB renders HRV changes indicating selective sympathetic inhibition of the heart without affecting vagal tone (Keim et al., 2025). Regarding LSB, early physiological studies indicated it could improve renal plasma flow and reduce renal vascular resistance (Solis-Herruzo et al., 1987). However, in the specific context of CKD, evidence remains largely confined to case reports and small pilot studies; for instance, Li et al. (2025) described significant improvements in CKD-associated pruritus following ultrasound-guided sympathetic nerve block, but RCTs targeting hypertensive CKD populations remain scarce. When compared to pharmacological conservative therapy, sympathetic nerve blocks offer a unique advantage for patients with RHTN who fail to respond to multiple drug regimens. Unlike pharmacotherapy, which often relies on patient compliance and may be limited by systemic side effects, nerve blocks provide a localized intervention.

### 4.2 Comparison with renal denervation (RDN)

RDN, as a representative interventional technique targeting the sympathetic nervous system, provides an important reference for evaluating the clinical status of ultrasound-guided blockade.

Clinical Evidence Evolution of RDN: The development of RDN has progressed through phases of optimism, skepticism, and renewed validation. Early Symplicity HTN-1 and HTN-2 trials demonstrated promising results, but the SYMPLICITY HTN-3 trial failed to meet its primary endpoint due to procedural and methodological limitations. However, recent technological advancements have re-established its efficacy. Landmark trials such as SPYRAL HTN-OFF MED (Townsend et al., 2017) and RADIANCE-HTN SOLO (Azizi et al., 2018) provided robust evidence that RDN significantly lowers blood pressure in the absence of antihypertensive medications. Subsequently, the RADIANCE-HTN TRIO trial (Azizi et al., 2021) confirmed its efficacy in patients with resistant hypertension on triple therapy.

While the KDIGO 2021 Clinical Practice Guideline for the Management of Blood Pressure in Chronic Kidney Disease (KDIGO Blood Pressure Work Group, 2021) has not yet incorporated RDN into its standard treatment algorithm, recent international hypertension guidelines have validated its use. Specifically, the 2023/2024 Guidelines from the European Society of Cardiology (ESC) and the European Society of Hypertension (ESH) now include RDN as a recommended interventional treatment for resistant hypertension (McEvoy et al., 2024). The absence of RDN recommendations in the KDIGO 2021 guideline reflects the limited inclusion of advanced CKD patients in early pivotal RDN trials and ongoing concerns regarding procedural safety in this vulnerable population. However, accumulating recent evidence demonstrates that RDN is a feasible and effective therapy in patients with chronic kidney disease, including those with moderate to severe CKD and kidney transplants. In the Global SYMPLICITY Registry, patients with moderate-to-severe CKD experienced clinically meaningful and durable blood pressure reductions without significant decline in eGFR or increase in renal adverse events over 3 years (Schlaich et al., 2025). A meta-analysis of 11 studies further supports the efficacy and safety of RDN in CKD populations, with sustained blood pressure reduction and preserved renal function over follow-up (Xia et al., 2021). Moreover, novel procedural approaches using CO₂ angiography and radial access have shown the feasibility of RDN even in severe CKD with minimal contrast exposure (Lo et al., 2024).

In contrast to RDN, ultrasound-guided sympathetic blockade (SGB/LSB) offers distinct characteristics that may be more suitable for CKD patients (see Table 1). Specifically: (1) Reversibility and Safety: While RDN causes long-lasting ablation of renal artery sympathetic nerves, it is not strictly permanent; evidence suggests that renal sympathetic nerves can undergo reinnervation over time (Booth et al., 2015). Furthermore, large human registries have demonstrated a highly favorable safety profile, with no direct procedural complications such as renal artery stenosis or thrombosis reported in contemporary long-term datasets (Schlaich et al., 2025). In contrast, SGB/LSB offers immediate pharmacological reversibility, allowing for a “test-and-treat” approach. (2) CKD Suitability: RDN requires arterial access, contrast agents, and fluoroscopy, posing risks of contrast-induced nephropathy and radiation exposure—factors particularly detrimental to CKD patients. Ultrasound-guided blockade is an extra-vascular procedure with zero contrast or radiation burden. (3) Additional Benefits: While RDN primarily targets blood pressure reduction, SGB/LSB provides pleiotropic benefits, including improvement in peripheral microcirculation (beneficial for diabetic foot ulcers) and stabilization of AVF function in hemodialysis patients.

**Table 1:** Comparison of ultrasound-guided sympathetic blockade (SGB/LSB/NCPB) vs. RDN.

Dimension	Ultrasound-guided SGB/LSB/NCPB	RDN
Mechanism	Neuromodulation: Temporary interruption of sympathetic transmission; systemic, regional, or splanchnic visceral regulation.	Ablation: Long-lasting interruption of renal sympathetic nerves; organ-specific targeting. (Note: Reinnervation occurs over time).
Evidence level	Emerging: Primarily small-scale RCTs, cohort studies, and case reports; lack of hard endpoint data.	Established: Supported by large-scale, sham-controlled RCTs (SPYRAL, RADIANCE); emerging evidence supports efficacy and safety in CKD cohorts.
Guideline status	Not Recommended: Not mentioned in standard hypertension or CKD guidelines for BP control.	Recommended (Conditional): Recommended by 2024 ESC/ESH guidelines for resistant hypertension; not yet included in KDIGO 2021 standard algorithm.
Invasiveness	Minimally Invasive: Local needle puncture; no arterial access, contrast, or radiation.	Interventional: Requires arterial puncture, angiography, and fluoroscopic/ultrasound guidance.
Reversibility	Reversible: Pharmacological effects wear off quickly; allows for “test-and-treat” strategy.	Long-lasting but not permanent: Anatomical and functional reinnervation occurs over time; provides sustained ablation.
Safety profile	Low Risk: Transient Horner’s syndrome, hoarseness, local hematoma; no renal toxicity.	Favorable Safety Profile: Large registries show no significant procedural complications (e.g., stenosis/thrombosis); primary risks relate to vascular access and contrast use.
Suitability for CKD	High: Safe for advanced CKD; preserves residual renal function; suitable for AVF maintenance.	Feasible: Recent evidence demonstrates efficacy and safety in moderate-to-severe CKD; contrast risks can be mitigated (e.g., CO₂ angiography), though caution is still advised in advanced stages.
Additional benefits	Multidimensional: Improves microcirculation, manages ischemic and visceral pain, regulates immunity.	Specific: Primarily focused on blood pressure reduction and organ protection secondary to BP control.

### 4.3 Changes in key clinical outcome indicators

Improvement in endothelial function serves as a critical marker of sympathetic blockade efficacy. Evidence suggests that SGB increases Flow-Mediated Dilation (FMD), a surrogate marker for endothelial health, by reducing norepinephrine spillover and oxidative stress (Hao et al., 2018). SGB significantly reduced serum cardiac troponin I and cardiac troponin T levels and mitigated the ST-segment depression and oxidative production levels. These findings suggest that SGB could have antioxidative effects against AMI, SGB could be an effective and safe method of local anesthesia to protect against cardiac damage due to oxidative stress (Wei et al., 2017). These changes indicate a reduction in afterload, which indirectly benefits renal perfusion. Direct renal protective effects have been observed through ultrasonographic and laboratory assessments. Ultrasound-guided LSB has been associated with a reduction in the Renal Resistive Index (RRI), suggesting improved renal microcirculation and reduced vascular resistance (Campese et al., 2006). In terms of laboratory markers, pilot studies have reported a decrease in the Urinary Albumin-to-Creatinine Ratio (UACR) following sympathetic blockade, likely due to reduced intraglomerular pressure. Although long-term data on the rate of eGFR decline is still accumulating, the acute improvement in renal hemodynamics suggests a potential for delaying CKD progression.

### 4.4 Subgroup analysis and special populations

CKD patients frequently suffer from diabetic nephropathy and peripheral vascular disease (PVD). For these patients, LSB is particularly beneficial. By inducing vasodilation in the lower extremities, LSB improves tissue perfusion, alleviates ischemic pain, and may indirectly facilitate glucose metabolism regulation by improving microcirculation. Patients with PVD have shown improved walking distances and ulcer healing rates, reducing the risk of amputation—a significant morbidity in the diabetic CKD population. Maintenance of Arteriovenous Fistula (AVF) Function in Hemodialysis Patients: A specific and clinically vital application in ESRD patients is the maintenance of AVF function. AVF dysfunction, often caused by vasospasm or stenosis due to high sympathetic tone, leads to dialysis inadequacy. Ultrasound-guided SGB has been utilized successfully to increase early patency rate and fistula maturation rate. Yildirim et al. (2006) demonstrated that SGB increased blood flow volume in the fistula vein, facilitating successful cannulation and prolonging fistula patency. This application highlights the role of sympathetic blockade as a vascular protection strategy in the hemodialysis cohort.

### 4.5 Precision medicine: identifying the ideal candidates

**Figure 2:**
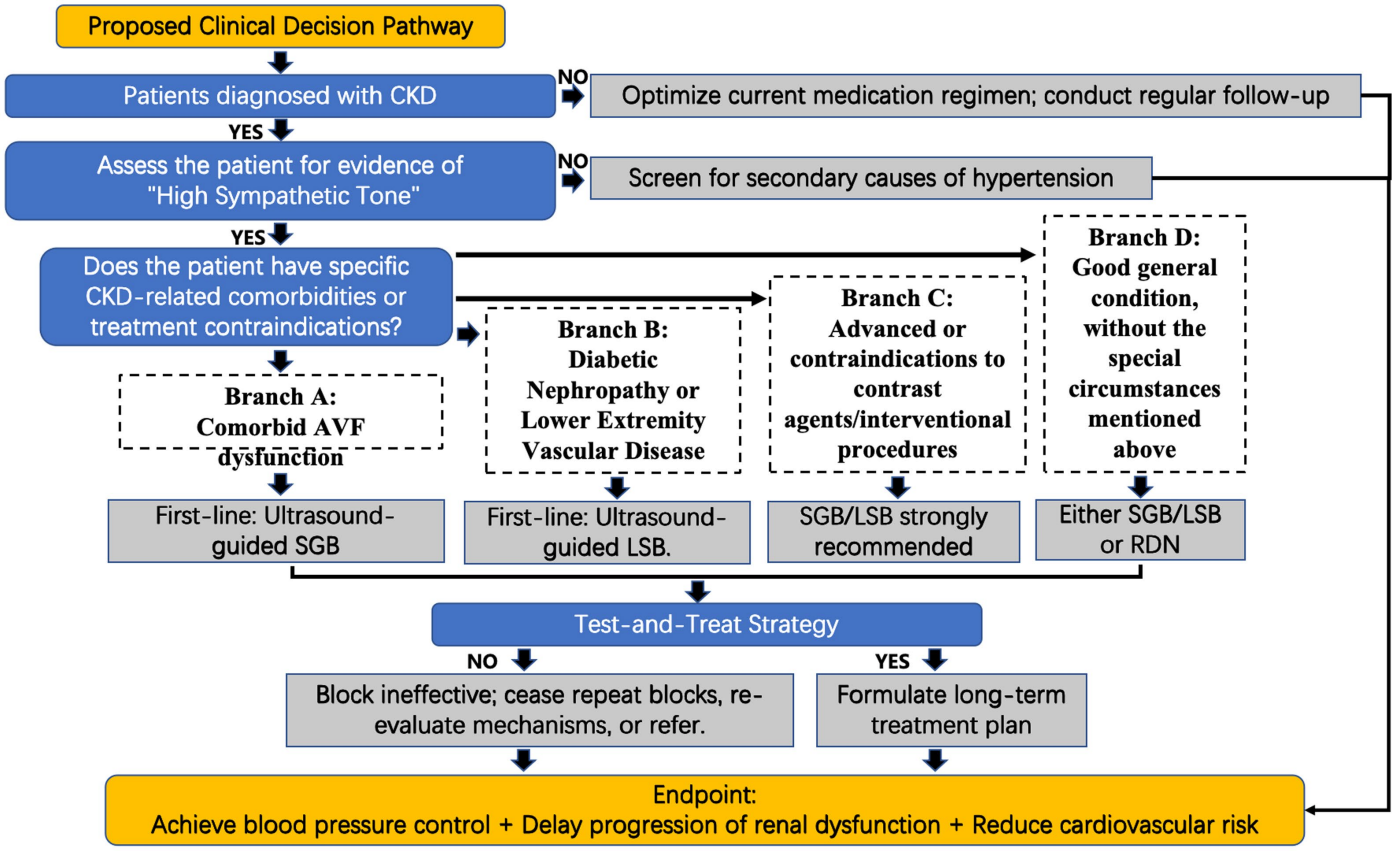
Proposed clinical decision pathway for ultrasound-guided sympathetic blockade in patients with hypertensive chronic kidney disease. This algorithm illustrates a precision medicine approach for managing resistant hypertension (RHTN) in chronic kidney disease (CKD) patients. The pathway initiates with screening for RHTN and confirming “high sympathetic tone” via biomarkers (e.g., HRV, MSNA, plasma norepinephrine). Treatment selection is stratified by specific comorbidities: stellate ganglion block (SGB) is prioritized for arteriovenous fistula (AVF) dysfunction, while lumbar sympathetic block (LSB) is indicated for peripheral vascular disease (PVD) or diabetic foot ulcers. For CKD patients with RHTN, both RDN and SGB/LSB are viable neuromodulation options. RDN offers sustained ablation and is conditionally recommended by recent ESC/ESH guidelines, with contrast risks manageable via strategies like CO_2_ angiography in severe CKD. SGB/LSB offers immediate reversibility and a “test-and-treat” approach, making it particularly suitable for patients requiring AVF maintenance, those with severe peripheral vascular disease, or when arterial access is contraindicated. The choice between RDN and SGB/LSB should be based on individual risk–benefit assessment, CKD stage, and specific comorbidities.

A critical barrier to clinical translation is patient selection. Not all CKD patients exhibit the same degree of sympathetic overactivity. Identifying “high sympathetic tone” patients is key to maximizing therapeutic efficacy (Figure 2). Biomarkers for Patient Screening: (1) Heart Rate Variability (HRV): As a non-invasive surrogate, reduced HRV (specifically low high-frequency power) indicates parasympathetic withdrawal and sympathetic dominance. Patients with low HRV may be ideal candidates for sympathetic modulation (Parati and Esler, 2012). (2) Muscle Sympathetic Nerve Activity (MSNA): The gold standard for quantifying sympathetic tone. Elevated baseline MSNA predicts a better antihypertensive response to sympathetic interventions. (3) Plasma Catecholamines: Elevated resting plasma norepinephrine levels can serve as a biochemical marker for hyperadrenergic states. (4) Test Block Strategy: The reversibility of ultrasound-guided blocks allows for a “test-and-treat” approach. A positive hemodynamic response to a single temporary block (e.g., significant BP reduction or improved RRI) can predict long-term efficacy, serving as a functional bioassay for patient selection.

## 5 Safety, limitations, and future perspectives

### 5.1 Safety and adverse reactions

Ultrasound-guided sympathetic nerve blocks are generally considered minimally invasive and safe procedures, but they are not devoid of risks. The most common adverse events are transient and self-limiting. For SGB, the occurrence of Horner’s syndrome (ptosis, miosis, and anhidrosis) is technically a sign of successful blockade rather than a complication, yet it can cause distress to patients if not adequately explained beforehand. Other transient side effects include hoarseness due to recurrent laryngeal nerve block and arm paresthesia. Local complications such as hematoma or infection at the injection site are rare, particularly with ultrasound guidance which allows for real-time visualization of vascular structures and needle trajectory.

A significant safety advantage of SGB and LSB over interventional therapies like RDN is their reversibility. RDN involves ablation of renal artery nerves, which results in long-lasting but not strictly permanent anatomical alteration, as reinnervation can occur over time (Booth et al., 2015). While early concerns existed regarding renal artery stenosis, contemporary large-scale registries have demonstrated a highly favorable safety profile without significant procedural complications such as stenosis or thrombosis (Schlaich et al., 2025). In contrast, chemical sympathetic blocks are pharmacologically reversible, and even if complications arise, they typically resolve within hours to days. This reversibility allows for a “test-and-treat” approach, where the patient’s response to a temporary block can be evaluated before considering more permanent interventions.

However, hemodynamic fluctuations remain a concern. In CKD patients with autonomic dysfunction, a sudden withdrawal of sympathetic tone can precipitate hypotension, particularly in those undergoing dialysis or on multiple antihypertensive agents. Vigilant monitoring of blood pressure post-procedure is essential, and dose adjustments of antihypertensive medications may be required to prevent symptomatic hypotension.

### 5.2 Current limitations

Despite promising findings, the clinical application of sympathetic blockade in hypertensive CKD is constrained by several limitations. First, there is a paucity of high-quality evidence. The current literature is dominated by small sample-sized studies, retrospective cohort analyses, and case reports. There is a distinct lack of large-scale, multi-center RCTs with long-term follow-up to definitively establish efficacy on hard endpoints such as cardiovascular mortality or progression to ESRD.

Second, the duration of the therapeutic effect remains a challenge. Sympathetic blocks using local anesthetics typically last for hours to days, whereas hypertension in CKD is a chronic condition requiring sustained management. The optimal frequency of repeated blocks has not been standardized; frequent injections increase the risk of procedure-related complications and reduce patient compliance. While neurolytic agents or radiofrequency ablation offer longer duration, they carry higher risks of neuritis and permanent nerve damage.

Third, patient selection lacks precision. It is currently unclear which specific subset of CKD patients derives the most benefit. Without established biomarkers, it is difficult to screen for “sensitive individuals” with high sympathetic tone, potentially diluting the observed treatment effect in clinical trials.

### 5.3 Future research directions

1. *Precision medicine and patient selection*: Future studies should focus on identifying patients who are most likely to benefit from sympathetic modulation. Precision medicine approaches utilizing HRV analysis or direct measurement of MSNA could serve as non-invasive and invasive tools, respectively, to quantify sympathetic overactivity. Patients with confirmed high sympathetic tone identified by these markers may be the ideal candidates for SGB or LSB. Additionally, factors such as sex differences in blood pressure regulation, linked to specific olfactory receptors, should be considered for patient stratification (Xu et al., 2024).
2. *Technical innovations*: To overcome the limitation of short duration, research into novel drug delivery systems is crucial. The development of long-acting local anesthetics or sustained-release formulations (e.g., liposomal bupivacaine) could extend the blockade duration, reducing the need for frequent injections. Furthermore, neuromodulation techniques such as Pulsed Radiofrequency (PRF) offer a middle ground between chemical blocks and ablation, providing longer pain relief and sympathetic inhibition without significant structural damage, representing a promising technical innovation in this field. Furthermore, novel organ-specific approaches to selectively target sensory afferents innervating the aortic arch or vagal pathways are being explored to refine neuromodulation strategies (Elsaafien et al., 2022; Baumer-Harrison et al., 2024).
3. *Combined therapy strategies*: Investigating the synergistic effects of sympathetic blocks with standard pharmacological treatments is a critical direction. Combining SGB or LSB with antihypertensive drugs may allow for lower drug dosages, thereby reducing pharmacological side effects while maintaining adequate blood pressure control. Additionally, exploring the combination of sympathetic blocks with RDN in RHTN cases warrants investigation to determine if multi-level sympathetic inhibition offers superior renal protection compared to single-modality therapy.

## 6 Conclusion

The pathophysiological link between autonomic imbalance and CKD progression is well-established. Sympathetic overactivity is not merely a compensatory mechanism for volume overload but a significant driver within the multifactorial pathophysiology of vascular injury, endothelial dysfunction, and accelerated renal decline in hypertensive CKD patients, as evidenced by robust animal models where neural ablation reverses these pathologies. In this context, ultrasound-guided sympathetic nerve block (SGB/LSB) offers a promising but still investigational therapeutic perspective by directly modulating the central and peripheral sympathetic nervous system. Current evidence suggests that sympathetic blockade exerts vasoprotective effects through a multi-dimensional mechanism: restoring the balance of the ANS, improving vascular endothelial function, and attenuating systemic inflammatory and oxidative stress responses. Unlike pharmacological therapies, which are often limited by systemic side effects and tolerance, and unlike RDN, which is irreversible and carries inherent vascular risks, ultrasound-guided sympathetic blockade provides a minimally invasive, reversible, and titratable option for regulating hemodynamics. It holds particular promise as an adjunctive strategy for patients with RHTN or those requiring vascular protection for AVFs. However, the transition of this technique from potential therapy to standard clinical practice requires robust validation. The current evidence base is limited by small sample sizes and heterogeneous study designs. Future research must prioritize large-scale, multi-center, prospective RCTs to definitively evaluate the long-term efficacy and safety of SGB and LSB in CKD populations. Furthermore, establishing standardized protocols for treatment frequency and identifying reliable biomarkers for patient selection are essential steps. Only through rigorous scientific validation can the role of sympathetic blockade be clearly defined within the comprehensive management of hypertensive CKD.

## References

[ref1] Ayad A. E. Agiza N. A. Elrifay A. H. Mortada A. M. Girgis M. Y. Varrassi G. (2024). Lumbar sympathetic block to treat CRPS in an 18-month-old girl: a breaking barriers case report and review of literature. Pain Ther. 13, 1325–1334. doi: 10.1007/s40122-024-00650-1, 39235698 PMC11544111

[ref2] Azizi M. Sanghvi K. Saxena M. Gosse P. Reilly J. P. Lobo M. D. . (2021). Ultrasound renal denervation for hypertension resistant to a triple medication pill (RADIANCE-HTN TRIO): a randomised, multicentre, single-blind, sham-controlled trial. Lancet 397, 2476–2486. doi: 10.1016/S0140-6736(21)00788-1, 34010611

[ref3] Azizi M. Schmieder R. E. Mahfoud F. Weber M. A. Daemen J. Davies J. . (2018). Endovascular ultrasound renal denervation to treat hypertension (RADIANCE-HTN SOLO): a multicentre, international, single-blind, randomised, sham-controlled trial. Lancet 391, 2335–2345. doi: 10.1016/S0140-6736(18)31082-1 29803590

[ref4] Bakris G. L. Agarwal R. Anker S. D. Pitt B. Ruilope L. M. Rossing P. . (2020). Effect of Finerenone on chronic kidney disease outcomes in type 2 diabetes. N. Engl. J. Med. 383, 2219–2229. doi: 10.1056/NEJMoa2025845, 33264825

[ref5] Baumer-Harrison C. Elsaafien K. Johnson D. N. Peñaloza Aponte J. D. de Araujo A. Patel S. . (2024). Alleviating hypertension by selectively targeting angiotensin receptor-expressing vagal sensory neurons. J. Neurosci. 44:e1154232023. doi: 10.1523/JNEUROSCI.1154-23.2023, 38242697 PMC10904025

[ref6] Becker B. K. Grady C. M. Markl A. E. Torres Rodriguez A. A. Pollock D. M. (2023). Elevated renal afferent nerve activity in a rat model of endothelin B receptor deficiency. Am J Physiol Renal Physiol 325, F235–F247. doi: 10.1152/ajprenal.00064.2023, 37348026 PMC10396274

[ref7] Booth L. C. Nishi E. E. Yao S. T. Ramchandra R. Lambert G. W. Schlaich M. P. . (2015). Reinnervation of renal afferent and efferent nerves at 5.5 and 11 months after catheter-based radiofrequency renal denervation in sheep. Hypertension 65, 393–400. doi: 10.1161/HYPERTENSIONAHA.114.04176, 25403610

[ref8] Campese V. M. Mitra N. Sandee D. (2006). Hypertension in renal parenchymal disease: why is it so resistant to treatment? Kidney Int. 69, 967–973. doi: 10.1038/sj.ki.5000177, 16528245

[ref9] Carmeliet P. (2000). Mechanisms of angiogenesis and arteriogenesis. Nat. Med. 6, 389–395. doi: 10.1038/74651, 10742145

[ref10] Chen Y. Q. Jin X. J. Liu Z. F. Zhu M. F. (2015). Effects of stellate ganglion block on cardiovascular reaction and heart rate variability in elderly patients during anesthesia induction and endotracheal intubation. J. Clin. Anesth. 27, 140–145. doi: 10.1016/j.jclinane.2014.06.012, 25559299

[ref11] Chien S. (2007). Mechanotransduction and endothelial cell homeostasis: the wisdom of the cell. Am. J. Physiol. Heart Circ. Physiol. 292, H1209–H1224. doi: 10.1152/ajpheart.01047.2006, 17098825

[ref12] Cousins M. J. Carr D. B. Horlocker T. T. Bridenbaugh P. O. (2012). Neural Blockade in Clinical Anesthesia and Pain Management. Philadelphia: Lippincott Williams.

[ref13] Damascelli B. Patelli G. Tichá V. Della Rocca F. Lattuada S. Sala C. . (2013). Catheter-based radiofrequency renal sympathetic denervation for resistant hypertension. J. Vasc. Interv. Radiol. 24, 632–639. doi: 10.1016/j.jvir.2013.01.491, 23622036

[ref14] Dinh Q. N. Drummond G. R. Sobey C. G. Chrissobolis S. (2014). Roles of inflammation, oxidative stress, and vascular dysfunction in hypertension. Biomed. Res. Int. 2014:406960. doi: 10.1155/2014/406960, 25136585 PMC4124649

[ref15] Elsaafien K. Harden S. W. Johnson D. N. Kimball A. K. Sheng W. Smith J. A. . (2022). A novel organ-specific approach to selectively target sensory afferents innervating the aortic arch. Front. Physiol. 13:841078. doi: 10.3389/fphys.2022.841078, 35399269 PMC8987286

[ref16] Elsaafien K. Kirchner M. K. Baumer-Harrison C. Tan Y. Johnson D. N. Vincent C. J. . (2026). Oxytocin and vasopressin cross talk within the brain increases blood pressure. Circ. Res. 138:e327322. doi: 10.1161/CIRCRESAHA.125.327322, 41616097 PMC12904227

[ref17] Elsaafien K. Kirchner M. K. Mohammed M. Eikenberry S. A. West C. Scott K. A. . (2021). Identification of novel cross-talk between the neuroendocrine and autonomic stress axes controlling blood pressure. J. Neurosci. 41, 4641–4657. doi: 10.1523/JNEUROSCI.0251-21.2021, 33858944 PMC8260250

[ref18] Fay K. S. Cohen D. L. (2021). Resistant hypertension in people with CKD: a review. Am. J. Kidney Dis. 77, 110–121. doi: 10.1053/j.ajkd.2020.04.017, 32712185

[ref19] Folkow B. (1982). Physiological aspects of primary hypertension. Physiol. Rev. 62, 347–504. doi: 10.1152/physrev.1982.62.2.347, 6461865

[ref20] Gao L. Wang W. Liu D. Zucker I. H. (2007). Exercise training normalizes sympathetic outflow by central antioxidant mechanisms in rabbits with pacing-induced chronic heart failure. Circulation 115, 3095–3102. doi: 10.1161/CIRCULATIONAHA.106.677989, 17548725

[ref21] Grassi G. Mark A. Esler M. (2015). The sympathetic nervous system alterations in human hypertension. Circ. Res. 116, 976–990. doi: 10.1161/CIRCRESAHA.116.303604, 25767284 PMC4367954

[ref22] Grassi G. Quarti-Trevano F. Seravalle G. Arenare F. Volpe M. Furiani S. . (2011). Early sympathetic activation in the initial clinical stages of chronic renal failure. Hypertension 57, 546–551. doi: 10.1161/HYPERTENSIONAHA.110.164780 21300663

[ref23] Guyatt G. H. Oxman A. D. Vist G. E. Kunz R. Falck-Ytter Y. Alonso-Coello P. . (2008). GRADE: an emerging consensus on rating quality of evidence and strength of recommendations. BMJ 336, 924–926. doi: 10.1136/bmj.39489.470347.AD, 18436948 PMC2335261

[ref24] Hao W. Yang R. Yang Y. Jin S. Li Y. Yuan F. . (2018). Stellate ganglion block ameliorates vascular calcification by inhibiting endoplasmic reticulum stress. Life Sci. 193, 1–8. doi: 10.1016/j.lfs.2017.12.002, 29208463

[ref25] Harrison D. G. Vinh A. Lob H. Madhur M. S. (2010). Role of the adaptive immune system in hypertension. Curr. Opin. Pharmacol. 10, 203–207. doi: 10.1016/j.coph.2010.01.006, 20167535 PMC2843787

[ref26] Higashi Y. Sasaki S. Nakagawa K. Matsuura H. Chayama K. Oshima T. (2001). Effect of obesity on endothelium-dependent, nitric oxide-mediated vasodilation in normotensive individuals. Am. J. Hypertens. 14, 1038–1045. doi: 10.1016/s0895-7061(01)02191-4, 11710783

[ref27] Hostetter T. H. Olson J. L. Rennke H. G. Venkatachalam M. A. Brenner B. M. (1981). Hyperfiltration in remnant nephrons: a potentially adverse response to renal ablation. Am. J. Phys. 241, F85–F93. doi: 10.1152/ajprenal.1981.241.1.F85, 7246778

[ref28] Intengan H. D. Schiffrin E. L. (2001). Vascular remodeling in hypertension: roles of apoptosis, inflammation, and fibrosis. Hypertension 38, 581–587. doi: 10.1161/hy0911.096249 11566935

[ref29] Janig W. Baron R. (2003). Complex regional pain syndrome: mystery explained? Lancet Neurol. 2, 687–697. doi: 10.1016/s1474-4422(03)00557-x, 14572737

[ref30] Jha V. Garcia-Garcia G. Iseki K. Li Z. Naicker S. Plattner B. . (2013). Chronic kidney disease: global dimension and perspectives. Lancet 382, 260–272. doi: 10.1016/S0140-6736(13)60687-X, 23727169

[ref31] Jiang W. Yang L. Zheng X. (2025). A prospective randomized clinical trial on the differential effect of left versus right stellate ganglion block on perioperative stress response. BMC Anesthesiol. 25:380. doi: 10.1186/s12871-025-03272-y, 40739539 PMC12312597

[ref32] Johns E. J. (2014). The neural regulation of the kidney in hypertension and renal failure. Exp. Physiol. 99, 289–294. doi: 10.1113/expphysiol.2013.072686, 23955311

[ref33] Joles J. A. Koomans H. A. (2004). Causes and consequences of increased sympathetic activity in renal disease. Hypertension 43, 699–706. doi: 10.1161/01.HYP.0000121881.77112.b1 14981063

[ref34] Katholi R. E. Winternitz S. R. Oparil S. (1981). Role of the renal nerves in the pathogenesis of one-kidney renal hypertension in the rat. Hypertension 3, 404–409. doi: 10.1161/01.hyp.3.4.404, 7030950

[ref35] KDIGO Blood Pressure Work Group (2021). KDIGO 2021 clinical practice guideline for the management of blood pressure in chronic kidney disease. Kidney Int. 99, S1–S87. doi: 10.1016/j.kint.2020.11.003 33637192

[ref36] Keim O. C. Raum R. C. Feldmann R. E. Kleinboehl D. Benrath J. (2025). Selective sympathetic action on heart rate variability after ultrasound-guided stellate ganglion block. Auton Neurosci 263:103367. doi: 10.1016/j.autneu.2025.103367 41352043

[ref37] Li Y. Chang J. Shi G. Zhang W. Wang H. Wei L. . (2023). Effects of stellate ganglion block on perimenopausal hot flashes: a randomized controlled trial. Front. Endocrinol. 14:1293358. doi: 10.3389/fendo.2023.1293358, 38089617 PMC10715304

[ref38] Li Y. Chen Y. Nie J. Huang X. Zhang W. (2025). Case report: sympathetic nerve block treat chronic kidney disease-associated pruritus. Front. Neurosci. 19:1529183. doi: 10.3389/fnins.2025.1529183, 40309654 PMC12040867

[ref39] Li D. P. Yang Q. Pan H. M. Pan H. L. (2008). Plasticity of pre- and postsynaptic GABAB receptor function in the paraventricular nucleus in spontaneously hypertensive rats. Am. J. Phys. Heart Circ. Phys. 295, H807–H815. doi: 10.1152/ajpheart.00259.2008, 18567709 PMC2519208

[ref40] Lo H. Y. Lee J. K. Lin Y. H. (2024). The feasibility, efficacy, and safety of RDN procedure using CO₂ angiography through radial artery in severe chronic kidney disease patients. Hypertens. Res. 47, 760–766. doi: 10.1038/s41440-023-01540-3, 38177288

[ref41] McEvoy J. W. McCarthy C. P. Bruno R. M. Brouwers S. Canavan M. D. Ceconi C. . (2024). 2024 ESC guidelines for the management of elevated blood pressure and hypertension. Eur. Heart J. 45, 3912–4018. doi: 10.1093/eurheartj/ehae178 39210715

[ref42] McMahon S. B. Koltzenburg M. Tracey I. Turk D. C. (2013). Wall and Melzack’s Textbook of Pain. Philadelphia: Elsevier Saunders.

[ref43] Montuoro S. Gentile F. Giannoni A. (2025). Neuroimmune cross-talk in heart failure. Cardiovasc. Res. 121, 550–567. doi: 10.1093/cvr/cvae236, 39498795

[ref44] Mulvaney S. W. McLean B. de Leeuw J. (2010). The use of stellate ganglion block in the treatment of panic/anxiety symptoms with combat-related post-traumatic stress disorder. Pain Pract. 10, 418–423. doi: 10.1111/j.1533-2500.2010.00373.x, 20412504

[ref45] Narouze S. (2010). Ultrasound-guided interventional procedures in pain management: evidence-based medicine. Reg. Anesth. Pain Med. 35, S55–S58. doi: 10.1097/AAP.0b013e3181d24658 20216026

[ref46] Parati G. Esler M. (2012). The human sympathetic nervous system: its relevance in hypertension and heart failure. Eur. Heart J. 33, 1058–1066. doi: 10.1093/eurheartj/ehs041, 22507981

[ref47] Raja S. N. Grabow T. S. (2002). Complex regional pain syndrome I (reflex sympathetic dystrophy). Anesthesiology 96, 1254–1260. doi: 10.1097/00000542-200205000-00031, 11981168

[ref48] Richter G. M. (2016). “Celiac plexus block and neurolysis,” in Image-Guided Interventions, eds. Mauro M. A. Murphy K. P. J. Thomson K. R. Venbrux A. C. Zollikofer C. L. (Amsterdam, Netherlands: Elsevier), 831–837.

[ref49] Roberts W. J. (1986). A hypothesis on the physiological basis for causalgia and related pains. Pain 24, 297–311. doi: 10.1016/0304-3959(86)90116-8, 3515292

[ref50] Sachdev A. H. Gress F. G. (2018). Celiac plexus block and neurolysis: a review. Gastrointest. Endosc. Clin. N. Am. 28, 579–586. doi: 10.1016/j.giec.2018.06.004 30241645

[ref51] Schiffrin E. L. (2001). Role of endothelin-1 in hypertension and vascular disease. Am. J. Hypertens. 14, 83S–89S. doi: 10.1016/s0895-7061(01)02074-x 11411770

[ref52] Schiffrin E. L. (2012). Vascular remodeling in hypertension: mechanisms and treatment. Hypertension 59, 367–374. doi: 10.1161/HYPERTENSIONAHA.111.187021 22203749

[ref53] Schlaich M. P. Mahfoud F. Böhm M. Narkiewicz K. Ruilope L. Williams B. . (2025). Renal denervation in patients with moderate to severe chronic kidney disease. Hypertension 82, 2252–2261. doi: 10.1161/HYPERTENSIONAHA.125.25470, 41159269 PMC12626611

[ref54] Shi P. Diez-Freire C. Jun J. Y. Qi Y. Katovich M. J. Li Q. . (2010). Brain microglial cytokines in neurogenic hypertension. Hypertension 56, 297–303. doi: 10.1161/HYPERTENSIONAHA.110.150409, 20547972 PMC2929640

[ref55] Shin J. Lee C. H. (2021). The roles of sodium and volume overload on hypertension in chronic kidney disease. Kidney Res Clin Pract 40, 542–554. doi: 10.23876/j.krcp.21.800, 34922428 PMC8685361

[ref56] Solis-Herruzo J. A. Duran A. Favela V. Castellano G. Madrid J. L. Muñoz-Yagüe M. T. . (1987). Effects of lumbar sympathetic block on kidney function in cirrhotic patients with hepatorenal syndrome. J. Hepatol. 5, 167–173. doi: 10.1016/s0168-8278(87)80569-x, 3693861

[ref57] Stanton-Hicks M. D. Burton A. W. Bruehl S. P. Carr D. B. Harden R. N. Hassenbusch S. J. . (2002). An updated interdisciplinary clinical pathway for CRPS: report of an expert panel. Pain Pract. 2, 1–16. doi: 10.1046/j.1533-2500.2002.02009.x, 17134466

[ref58] Stenvinkel P. (2010). Chronic kidney disease: a public health priority and harbinger of premature cardiovascular disease. J. Intern. Med. 268, 456–467. doi: 10.1111/j.1365-2796.2010.02269.x, 20809922

[ref59] Townsend R. R. Mahfoud F. Kandzari D. E. Kario K. Pocock S. Weber M. A. . (2017). Catheter-based renal denervation in patients with uncontrolled hypertension in the absence of antihypertensive medications (SPYRAL HTN-OFF MED): a randomised, sham-controlled, proof-of-concept trial. Lancet 390, 2160–2170. doi: 10.1016/S0140-6736(17)32281-X, 28859944

[ref60] Tracey K. J. (2007). Physiology and immunology of the cholinergic antiinflammatory pathway. J. Clin. Invest. 117, 289–296. doi: 10.1172/JCI30555, 17273548 PMC1783813

[ref61] Treede R. D. Davis K. D. Campbell J. N. Raja S. N. (1992). The plasticity of cutaneous hyperalgesia during sympathetic ganglion blockade in patients with neuropathic pain. Brain 115, 607–621. doi: 10.1093/brain/115.2.607, 1606484

[ref62] Vink E. E. de Jager R. L. Blankestijn P. J. (2013). Sympathetic hyperactivity in chronic kidney disease: pathophysiology and (new) treatment options. Curr. Hypertens. Rep. 15, 95–101. doi: 10.1007/s11906-013-0328-5, 23354877

[ref3003] Wei N. Chi M. Deng L. Wang G. (2017). Antioxidation role of different lateral stellate ganglion block in isoproterenol-induced acute myocardial ischemia in rats. Regional Anesthesia and Pain Medicine, 42, 588–594. doi: 10.1097/AAP.0000000000000647 28820802

[ref63] Xia M. Liu T. Chen D. Huang Y. (2021). Efficacy and safety of renal denervation for hypertension in patients with chronic kidney disease: a meta-analysis. Int. J. Hyperth. 38, 732–742. doi: 10.1080/02656736.2021.1916100, 33908329

[ref64] Xu J. Choi R. Gupta K. Warren H. R. Santhanam L. Pluznick J. L. (2024). An evolutionarily conserved olfactory receptor is required for sex differences in blood pressure. Sci. Adv. 10:eadk1487. doi: 10.1126/sciadv.adk1487, 38507492 PMC10954203

[ref65] Xu J. Moore B. N. Pluznick J. L. (2022). Short-chain fatty acid receptors and blood pressure regulation: council on hypertension mid-career award for research excellence 2021. Hypertension 79, 2127–2137. doi: 10.1161/HYPERTENSIONAHA.122.18558, 35912645 PMC9458621

[ref66] Yildirim V. Doganci S. Yanarates O. Saglam M. Kuralay E. Cosar A. . (2006). Does preemptive stellate ganglion blockage increase the patency of radiocephalic arteriovenous fistula? Scand. Cardiovasc. J. 40, 380–384. doi: 10.1080/14017430600913207, 17118830

